# Journalism as a Teacher

**Published:** 1883-07-20

**Authors:** 


### *JOURNALISM AS A TEACHER.*

Many centuries ago Bion (not Byron) wrote "Know thyself," and in this terse and laconic admonition, we are taught the importance of a thorough knowledge of both man's physical and psychological conditions—that the science of man is the greatest of all, for it is the science of the body, that home of the soul—of the material that will one day be remodeled and transfigured with the glory of the immaterial, undimned through the cycles of neverending ages.

It can no longer be said that the science of man lies shrouded in apathy, and is behind all other arts of the age. It is already aroused from its slumbers, and shaking from its robes the dust of centuries, steps to the front with a confidence and courage born of a truer and nobler emulation, and a consciousness of power to keep pace with other professional arts.

We repeat here that our progress in the profession largely depends upon our instructors in medical science and literature.

What we teach in medical schools, only reach a limited number under immediate educational supervision; but what we write and publish in our Journals will be, perhaps, desseminated throughout the medical world. As instructors in the editorial chair, we are regarded as the oracles of the profession. Not only the student of medicine, but the great mass of the profession, look to us for knowledge, counsel and guidance in the true and safe path they wish to tread. As medical editors, we must supply them with the wisdom that will make them wise in the revelations of successful, progressive medicine.

To do this, we must first have a thorough understanding of our position, and then labor to reach the great heights in the profession, so we may be fitted to lead. those in the ranks up to the same lofty altitude. As one of the honored presidents of the American Medical Association has said, when we enlighten the professional readers, we develop in the minds of those who have passed the noviate of their studies, a desire for a higher and fuller attainment in their future profession.

And further: We can also awaken the country practitioners throughout the land to a sense of their equal responsibilities with those of cities in carrying on the work of advanced knowledge in medicine.

We must so educate the tone and thought of the professional masses that a physician will feel he has no right to claim the confidence of the people in his ability to treat disease, or to expect their support unless he is fully accomplished in the "protracted study and high requirements for the doctorate."

Let us have more valuable additions to Medicine and Surgery through our journalism, and more original, earnest and patient investigation of all the arts and sciences that come within our province; and above all, let us have a deeper study of nature's laws as adapted to the principles of medicine.

We can continually develop new facts bearing upon the structure, functions and diseases of the human body, and "every stone added to the temple of knowledge" will contribute to build a solid and enduring structure upon which the profession may rest in confidence and security.

The character of our journalism should be frank, courteous, instructive and communicative. If we learn or discover a truth that will benefit the profession, let us give it without reserve, as common property. We will then banish ungenerous feelings towards our professional brethren, and crush all petty jealousies that must, and that do, damage the progressive spirit of medicine, and that lower the dignity and high tone the editorial corps should always maintain.

Without unanimity of feeling, we cannot foster and facilitate frank and friendly intercourse, and without concert of action, we cannot impress upon the ranks of the profession a knowledge of the power there is in combined forces to bring about the change in the requirements for those attainments in medicine we so earnestly desire.

Let us also place a just estimate upon our medical schools. If they are worthy of eulogy—if they have a high character in all their features, let us give them our hearty commendation, and through our journalism place them before the profession in their true light and superior position. To encourage the emulation of this high character in our institutions, we should condemn all dishonorable means employed to increase the number of students, and any methods that will give our medical colleges a false position.

The eyes of the profession are open, and the time is fast approaching when colleges will be estimated like individuals, by the old adage, "Show me with whom thou goeth and I will tell thee what thou doeth." T. S. P.

*Blue Ridge Springs, Virginia, July* 16 1883.

